# Supplementary material for: Mechanisms governing the pioneering and redistribution capabilities of the non-classical pioneer PU.1
Source: Nat Commun. 2020 Jan 21;11:402. doi: 10.1038/s41467-019-13960-2 (PMC6972792; doi:10.1038/s41467-019-13960-2)
Supplement: Supplementary file 7 — Source data [file 41467_2019_13960_MOESM7_ESM.zip › Source_Data/FigureS2/FigureS2B_MotifScanOutput/KmeansCluster4/homerResults.html]

/misc/data/analysis/project\_PU1/CTV1/motifs/basic/PU1\_Kmeans14.4/final - Homer de novo Motif Results


# Homer *de novo* Motif Results (/misc/data/analysis/project\_PU1/CTV1/motifs/basic/PU1\_Kmeans14.4/final)

Known Motif Enrichment Results  
Gene Ontology Enrichment Results  
If Homer is having trouble matching a motif to a known motif, try copy/pasting the matrix file into
STAMP  
More information on motif finding results: HOMER
| Description of Results
| Tips
  
Total target sequences = 4515  
Total background sequences = 41782  
\* - possible false positive  

|  |  |  |  |  |  |  |  |  |
| --- | --- | --- | --- | --- | --- | --- | --- | --- |
| Rank | Motif | P-value | log P-pvalue | % of Targets | % of Background | STD(Bg STD) | Best Match/Details | Motif File |
| 1 | A C G T A T G C C T G A A T G C A C G T A C G T A G T C A G T C G A C T A G T C G C A T G A C T G C A T A G C T | 1e-2368 | -5.453e+03 | 87.60% | 13.26% | 32.2bp (73.7bp) | SpiB(ETS)/OCILY3-SPIB-ChIP-Seq(GSE56857)/Homer(0.952) More Information \| Similar Motifs Found | motif file (matrix) |
| 2 | A T G C A G C T A G C T A G T C A G C T A G C T A G T C | 1e-620 | -1.428e+03 | 73.91% | 32.89% | 49.1bp (84.7bp) | TRA2(RRM)/Drosophila\_melanogaster-RNCMPT00078-PBM/HughesRNA(0.847) More Information \| Similar Motifs Found | motif file (matrix) |
| 3 | A G T C A G C T A G C T A G T C G A T C G A C T A T G C G A C T A C G T G A T C G A T C G A C T A G T C | 1e-358 | -8.257e+02 | 38.83% | 13.00% | 50.4bp (88.5bp) | PB0058.1\_Sfpi1\_1/Jaspar(0.730) More Information \| Similar Motifs Found | motif file (matrix) |
| 4 | A G T C C G T A T A C G C G A T A C G T A G T C A G C T T G A C | 1e-327 | -7.552e+02 | 31.07% | 9.00% | 47.3bp (74.1bp) | SpiB(ETS)/OCILY3-SPIB-ChIP-Seq(GSE56857)/Homer(0.733) More Information \| Similar Motifs Found | motif file (matrix) |
| 5 | C G T A C G T A A C T G C G T A C T A G A C T G C G T A | 1e-295 | -6.797e+02 | 28.15% | 8.08% | 51.4bp (77.6bp) | Unknown4/Arabidopsis-Promoters/Homer(0.757) More Information \| Similar Motifs Found | motif file (matrix) |
| 6 | T C G A C T G A C T G A C G T A C G T A T C A G A T G C C G T A A T C G G T C A C G T A T A C G G A C T | 1e-186 | -4.286e+02 | 13.67% | 2.84% | 40.6bp (75.5bp) | SPI1/MA0080.4/Jaspar(0.780) More Information \| Similar Motifs Found | motif file (matrix) |
| 7 | C A T G T G C A C G T A T C A G C T G A A C T G T C A G T G C A G C T A T C A G T C G A C A T G A C T G G T C A | 1e-176 | -4.059e+02 | 15.15% | 3.73% | 52.6bp (76.4bp) | SPIB/MA0081.1/Jaspar(0.646) More Information \| Similar Motifs Found | motif file (matrix) |
| 8 | A G C T A C G T C G A T C T A G T G C A C T A G A T C G C G T A G T C A A T C G | 1e-118 | -2.727e+02 | 10.41% | 2.57% | 48.0bp (74.5bp) | SPIB/MA0081.1/Jaspar(0.795) More Information \| Similar Motifs Found | motif file (matrix) |
| 9 | A T G C G T C A T G A C C G A T G A C T T G A C G A T C C G A T A C T G C T A G A G C T | 1e-109 | -2.524e+02 | 10.99% | 3.05% | 55.1bp (70.6bp) | Eip74EF/dmmpmm(Pollard)/fly(0.789) More Information \| Similar Motifs Found | motif file (matrix) |
| 10 | A G C T C A G T T A G C G A T C G A C T A T G C G T A C G A C T G T A C G T A C G A C T A T G C G T A C G C A T | 1e-104 | -2.399e+02 | 11.78% | 3.62% | 53.8bp (77.1bp) | PF10\_0068(RRM)/Plasmodium\_falciparum-RNCMPT00199-PBM/HughesRNA(0.679) More Information \| Similar Motifs Found | motif file (matrix) |
| 11 | C T G A T C G A T G C A C T G A T G C A C G T A C A T G C A T G T C A G T G C A G C T A T G A C G A C T | 1e-79 | -1.825e+02 | 6.42% | 1.44% | 46.7bp (72.2bp) | NDD1(MacIsaac)/Yeast(0.726) More Information \| Similar Motifs Found | motif file (matrix) |
| 12 | A C T G A G T C A C G T A C G T A G T C A T G C A G C T | 1e-71 | -1.651e+02 | 40.38% | 27.28% | 55.1bp (72.7bp) | GCR2(MacIsaac)/Yeast(0.939) More Information \| Similar Motifs Found | motif file (matrix) |
| 13 | G A C T T G A C A G C T A G T C T C G A G A C T T C A G C T G A C T A G G T C A C G T A A G T C C G A T | 1e-66 | -1.535e+02 | 3.94% | 0.61% | 43.1bp (71.5bp) | GFX(?)/Promoter/Homer(0.718) More Information \| Similar Motifs Found | motif file (matrix) |
| 14 | A C G T A T C G A G T C A G C T A C T G A C G T A G C T | 1e-59 | -1.375e+02 | 24.92% | 14.99% | 55.9bp (70.6bp) | ACE2/ACE2\_YPD/2-SWI5(Harbison)/Yeast(0.814) More Information \| Similar Motifs Found | motif file (matrix) |
| 15 | C T A G A G C T C T A G C T G A T A C G C G T A C G T A A G T C A C G T A G C T C G T A A G T C | 1e-56 | -1.306e+02 | 2.77% | 0.32% | 53.9bp (57.4bp) | Su(H)/dmmpmm(Papatsenko)/fly(0.696) More Information \| Similar Motifs Found | motif file (matrix) |
| 16 | A C T G A C T G C T G A A C T G A C T G C G T A C T A G A C T G C G T A | 1e-56 | -1.291e+02 | 8.06% | 2.90% | 48.2bp (95.2bp) | SRSF1(RRM)/Homo\_sapiens-RNCMPT00163-PBM/HughesRNA(0.850) More Information \| Similar Motifs Found | motif file (matrix) |
| 17 | C G T A G A T C G T C A A G T C G A C T A C G T A G C T G C A T T C G A G T C A C G T A A G T C G T A C C T G A | 1e-55 | -1.273e+02 | 2.10% | 0.14% | 27.9bp (73.8bp) | AT1G69570(C2C2dof)/col-AT1G69570-DAP-Seq(GSE60143)/Homer(0.665) More Information \| Similar Motifs Found | motif file (matrix) |
| 18 | A G T C G A T C G C A T A T G C G C T A C T A G T C A G T C G A G T C A T C G A A G T C G A C T C G A T G T C A | 1e-54 | -1.253e+02 | 3.94% | 0.78% | 71.1bp (65.6bp) | EWS:FLI1-fusion(ETS)/SK\_N\_MC-EWS:FLI1-ChIP-Seq(SRA014231)/Homer(0.707) More Information \| Similar Motifs Found | motif file (matrix) |
| 19 | C T G A C G T A C G T A A C T G A C G T A C T G A C G T A C T G | 1e-53 | -1.238e+02 | 6.51% | 2.08% | 42.6bp (75.0bp) | RBM24(RRM)/Homo\_sapiens-RNCMPT00184-PBM/HughesRNA(0.807) More Information \| Similar Motifs Found | motif file (matrix) |
| 20 | A C G T C T A G T C G A A G C T C G A T C T A G T C A G G T C A A G C T A G T C C G T A A G C T A T C G | 1e-51 | -1.186e+02 | 2.28% | 0.22% | 73.0bp (65.0bp) | PH0014.1\_Cphx/Jaspar(0.830) More Information \| Similar Motifs Found | motif file (matrix) |
| 21 | A C T G C T A G G T A C C G T A C A T G C G A T A T C G A T G C C G T A A C T G T C A G C T G A A C T G G C T A | 1e-51 | -1.178e+02 | 5.63% | 1.67% | 53.3bp (68.8bp) | SRSF1(RRM)/Homo\_sapiens-RNCMPT00163-PBM/HughesRNA(0.589) More Information \| Similar Motifs Found | motif file (matrix) |
| 22 | C G T A C T G A A G C T A G T C C T G A G C A T C T A G C T A G G A T C C T A G T C A G G T C A T C G A | 1e-50 | -1.167e+02 | 3.15% | 0.52% | 64.1bp (70.0bp) | HAT1/MA1024.1/Jaspar(0.688) More Information \| Similar Motifs Found | motif file (matrix) |
| 23 | A G T C A G C T G A T C G T C A A G T C G A C T T C G A G A C T A G T C G T C A A G T C C T A G C T G A | 1e-50 | -1.166e+02 | 2.57% | 0.32% | 49.3bp (75.8bp) | FZF1/MA0298.1/Jaspar(0.682) More Information \| Similar Motifs Found | motif file (matrix) |
| 24 | C T A G C T G A A C G T C G A T C A T G C G A T G C T A T C G A A C T G C G A T A G C T | 1e-45 | -1.055e+02 | 2.52% | 0.35% | 71.1bp (61.8bp) | PUM(PUF)/Drosophila\_melanogaster-RNCMPT00105-PBM/HughesRNA(0.679) More Information \| Similar Motifs Found | motif file (matrix) |
| 25 | C T G A A C T G G T C A C T G A C A T G A C T G C G T A C T A G T C G A G T C A C T G A T A C G C T G A A C T G | 1e-44 | -1.034e+02 | 3.74% | 0.87% | 44.7bp (82.5bp) | Tb\_0230(RRM)/Trypanosoma\_brucei-RNCMPT00230-PBM/HughesRNA(0.640) More Information \| Similar Motifs Found | motif file (matrix) |
| 26 | T C A G G T A C T C G A C G A T C T A G C T A G C T A G C T A G C T A G G T C A C G T A G T C A A G C T | 1e-42 | -9.779e+01 | 1.93% | 0.20% | 58.3bp (51.3bp) | RO3G\_00049(RRM)/Rhizopus\_oryzae-RNCMPT00205-PBM/HughesRNA(0.676) More Information \| Similar Motifs Found | motif file (matrix) |
| 27 | A T C G A C G T T C A G C G T A A C T G A C T G A G T C G T A C A C G T G A T C | 1e-37 | -8.624e+01 | 3.79% | 1.05% | 70.5bp (71.0bp) | JUND/MA0491.1/Jaspar(0.609) More Information \| Similar Motifs Found | motif file (matrix) |
| 28 | G T A C A G T C C G A T A G C T A G T C C G A T A G T C G A C T A C G T A C G T A G T C G A C T A C G T A T G C | 1e-37 | -8.617e+01 | 3.81% | 1.06% | 43.5bp (75.1bp) | BPC1(BBRBPC)/colamp-BPC1-DAP-Seq(GSE60143)/Homer(0.732) More Information \| Similar Motifs Found | motif file (matrix) |
| 29 | A T G C A G T C T A G C G T A C T A G C T C G A A C G T C T A G T G C A C G A T | 1e-33 | -7.774e+01 | 4.05% | 1.28% | 66.1bp (70.0bp) | Tv\_0259(RRM)/Trichomonas\_vaginalis-RNCMPT00259-PBM/HughesRNA(0.765) More Information \| Similar Motifs Found | motif file (matrix) |
| 30 | G T A C G T C A T A G C C G A T A C G T G T A C G T A C A G C T G C A T C T G A C G T A C G T A C T G A | 1e-32 | -7.539e+01 | 1.57% | 0.18% | 40.5bp (63.2bp) | Bcl6/MA0463.1/Jaspar(0.672) More Information \| Similar Motifs Found | motif file (matrix) |
| 31 | C G T A A G T C G T A C C G T A A C T G C G T A A C G T A T G C A C G T | 1e-31 | -7.175e+01 | 5.69% | 2.35% | 49.9bp (64.1bp) | GATA14(C2C2gata)/col-GATA14-DAP-Seq(GSE60143)/Homer(0.895) More Information \| Similar Motifs Found | motif file (matrix) |
| 32 | A G T C C G A T A G T C G A C T A G T C A C G T A G T C C G A T A G T C A G T C A C G T C T A G A G T C | 1e-29 | -6.893e+01 | 1.35% | 0.14% | 29.7bp (59.4bp) | SeqBias: GA-repeat(0.776) More Information \| Similar Motifs Found | motif file (matrix) |
| 33 | A G T C A G T C C G T A A G T C C T A G A G C T A C T G C T A G A G C T | 1e-28 | -6.614e+01 | 10.34% | 5.77% | 51.9bp (66.7bp) | Mycn/MA0104.3/Jaspar(0.888) More Information \| Similar Motifs Found | motif file (matrix) |
| 34 | G T A C A T G C C G A T A C G T G A T C A C G T A C T G A G T C A G T C C G T A | 1e-26 | -6.160e+01 | 2.64% | 0.72% | 57.1bp (66.0bp) | REF2(RRM)/Drosophila\_melanogaster-RNCMPT00059-PBM/HughesRNA(0.699) More Information \| Similar Motifs Found | motif file (matrix) |
| 35 | A G C T G C T A C G T A C G T A G T C A A G T C G A T C G T C A A G C T A G T C | 1e-23 | -5.520e+01 | 3.74% | 1.42% | 53.0bp (64.8bp) | grh/dmmpmm(Pollard)/fly(0.744) More Information \| Similar Motifs Found | motif file (matrix) |
| 36 | C T A G G T A C A G T C C G T A A G C T C T A G A G C T A C T G C G T A T C G A T C A G | 1e-23 | -5.506e+01 | 2.30% | 0.62% | 46.7bp (70.6bp) | INO4/INO4\_YPD/4-INO4,37-INO2(Harbison)/Yeast(0.827) More Information \| Similar Motifs Found | motif file (matrix) |
| 37 | A C T G C T A G C G T A A T C G C T A G A C G T C T A G C G T A A C G T A C G T | 1e-23 | -5.484e+01 | 1.82% | 0.39% | 75.5bp (69.5bp) | PL0011.1\_hlh-2::hlh-4/Jaspar(0.650) More Information \| Similar Motifs Found | motif file (matrix) |
| 38 | A G T C G C A T C T A G G T A C C G A T C A T G A G T C G A C T C A T G A T G C | 1e-22 | -5.241e+01 | 11.07% | 6.79% | 57.2bp (72.1bp) | Adf1/dmmpmm(Bergman)/fly(0.745) More Information \| Similar Motifs Found | motif file (matrix) |
| 39 | A T C G A G T C A C G T A C G T T G A C A G T C G A T C A C G T C G T A C G T A A G C T C G A T | 1e-22 | -5.189e+01 | 2.37% | 0.68% | 50.7bp (71.8bp) | GCR2/MA0305.1/Jaspar(0.735) More Information \| Similar Motifs Found | motif file (matrix) |
| 40 | C G A T A C T G A C G T C G T A C G A T A G T C A C G T A C T G A G T C C G A T C T G A | 1e-22 | -5.167e+01 | 7.22% | 3.86% | 57.9bp (70.2bp) | grh/dmmpmm(Papatsenko)/fly(0.657) More Information \| Similar Motifs Found | motif file (matrix) |
| 41 | C T G A C T A G A C T G A T G C C G T A A C T G A C T G C T G A C G T A A T C G A G C T A G C T C G T A | 1e-21 | -4.939e+01 | 1.11% | 0.15% | 47.4bp (54.8bp) | ETS1(ETS)/Jurkat-ETS1-ChIP-Seq(GSE17954)/Homer(0.770) More Information \| Similar Motifs Found | motif file (matrix) |
| 42 | A G C T C G A T A G T C A G T C A G T C A C T G G C T A A T C G A C T G | 1e-21 | -4.853e+01 | 2.83% | 0.98% | 74.0bp (73.0bp) | PUT3/MA0358.1/Jaspar(0.729) More Information \| Similar Motifs Found | motif file (matrix) |
| 43 | A C T G C T A G A C G T C G T A C T A G A C G T A C T G C G T A | 1e-21 | -4.851e+01 | 5.80% | 2.92% | 55.7bp (65.1bp) | At5g04390(C2H2)/col200-At5g04390-DAP-Seq(GSE60143)/Homer(0.787) More Information \| Similar Motifs Found | motif file (matrix) |
| 44 | A C T G A C T G A C G T C T A G C G T A A C G T A C G T C T A G A C T G | 1e-20 | -4.812e+01 | 1.57% | 0.33% | 73.7bp (66.1bp) | HAP4/HAP4\_YPD/1-GZF3,11-HAP4(Harbison)/Yeast(0.819) More Information \| Similar Motifs Found | motif file (matrix) |
| 45 | A G T C C T G A A G T C C T A G C T A G G A T C G C T A T C A G | 1e-20 | -4.792e+01 | 11.65% | 7.43% | 60.2bp (67.7bp) | Unknown-ESC-element(?)/mES-Nanog-ChIP-Seq(GSE11724)/Homer(0.698) More Information \| Similar Motifs Found | motif file (matrix) |
| 46 | C T G A A C T G G T A C C G T A G A C T C T G A C G A T G T A C A G C T A C G T C G T A A G T C | 1e-19 | -4.573e+01 | 1.13% | 0.17% | 49.0bp (64.4bp) | MEX-5(Znf)/Caenorhabditis\_elegans-RNCMPT00039-PBM/HughesRNA(0.616) More Information \| Similar Motifs Found | motif file (matrix) |
| 47 | C G T A T C G A A G T C A T G C C T G A A G T C T G C A | 1e-19 | -4.488e+01 | 32.23% | 25.78% | 59.0bp (67.1bp) | RUNX1(Runt)/Jurkat-RUNX1-ChIP-Seq(GSE29180)/Homer(0.845) More Information \| Similar Motifs Found | motif file (matrix) |
| 48 | C G T A A G T C A C G T A G T C C G T A A G T C A C G T | 1e-18 | -4.232e+01 | 7.42% | 4.29% | 53.6bp (72.0bp) | STZ(C2H2)/colamp-STZ-DAP-Seq(GSE60143)/Homer(0.831) More Information \| Similar Motifs Found | motif file (matrix) |
| 49 | C T G A G T A C G A T C A C G T A C T G A C T G A C G T C T A G A T C G A C T G C T G A C T A G C T A G | 1e-17 | -4.088e+01 | 0.89% | 0.11% | 77.3bp (82.5bp) | schlank/MA0193.1/Jaspar(0.662) More Information \| Similar Motifs Found | motif file (matrix) |
| 50 | A C T G G A C T T C G A A T G C A C G T T C G A C G T A A C G T G A C T C G T A | 1e-17 | -4.014e+01 | 2.08% | 0.67% | 57.9bp (57.0bp) | PB0096.1\_Zfp187\_1/Jaspar(0.781) More Information \| Similar Motifs Found | motif file (matrix) |
| 51 | A C T G A G T C T G C A A C T G C G T A C G T A A G T C | 1e-17 | -3.938e+01 | 6.67% | 3.81% | 54.9bp (69.2bp) | Ot\_0263(RRM)/Ostreococcus\_tauri-RNCMPT00263-PBM/HughesRNA(0.774) More Information \| Similar Motifs Found | motif file (matrix) |
| 52 | A G C T G T A C G C A T G A C T G T C A T A G C G A T C G T C A C G A T C A T G T A C G G A C T C T G A | 1e-15 | -3.494e+01 | 0.33% | 0.00% | 43.6bp (0.0bp) | X-box(HTH)/NPC-H3K4me1-ChIP-Seq(GSE16256)/Homer(0.708) More Information \| Similar Motifs Found | motif file (matrix) |
| 53 | C T G A C T G A A T C G C G T A T G C A A G T C C G A T C A T G T A G C C G A T T A C G | 1e-14 | -3.238e+01 | 3.03% | 1.38% | 62.5bp (68.7bp) | PB0047.1\_Myf6\_1/Jaspar(0.751) More Information \| Similar Motifs Found | motif file (matrix) |
| 54 | A C G T A C T G A G C T C G T A G T A C A C G T G T C A C T G A C G A T A C G T A C G T | 1e-13 | -3.192e+01 | 0.71% | 0.09% | 49.4bp (58.4bp) | PB0096.1\_Zfp187\_1/Jaspar(0.789) More Information \| Similar Motifs Found | motif file (matrix) |
| 55 | T A C G T C G A C T G A A T C G C G A T C T G A C A T G T C A G C G T A C T G A C T A G G T C A | 1e-13 | -3.153e+01 | 0.73% | 0.10% | 51.5bp (41.1bp) | At3g60580(C2H2)/col-At3g60580-DAP-Seq(GSE60143)/Homer(0.687) More Information \| Similar Motifs Found | motif file (matrix) |
| 56 | A C G T A G T C C T G A A C G T A C T G C T G A A C T G | 1e-13 | -3.035e+01 | 14.17% | 10.43% | 58.8bp (68.4bp) | MAFG::NFE2L1/MA0089.1/Jaspar(0.767) More Information \| Similar Motifs Found | motif file (matrix) |
| 57 | A C T G G T C A C G T A C T A G A C T G C G T A A C T G C G T A T C G A A C T G A C T G C G T A A T C G C G T A | 1e-12 | -2.833e+01 | 0.84% | 0.17% | 66.0bp (71.0bp) | RBM5(RRM)/Homo\_sapiens-RNCMPT00154-PBM/HughesRNA(0.650) More Information \| Similar Motifs Found | motif file (matrix) |
